# Floral ultrastructure of two Brazilian aquatic-epiphytic bladderworts: *Utricularia cornigera* Studnička and *U. nelumbifolia* Gardner (Lentibulariaceae)

**DOI:** 10.1007/s00709-016-0956-0

**Published:** 2016-03-05

**Authors:** Bartosz J. Płachno, Małgorzata Stpiczyńska, Kevin L. Davies, Piotr Świątek, Vitor Fernandes Oliveira de Miranda

**Affiliations:** 1 Department of Plant Cytology and Embryology, Jagiellonian University in Kraków, 9 Gronostajowa St., 30-387 Kraków, Poland; 2 Faculty of Biology, University of Warsaw, Botanic Garden Al. Ujazdowskie 4, 00-478 Warsaw, Poland; 3 School of Earth and Ocean Sciences, Cardiff University, Main Building, Park Place, Cardiff, CF10 3AT UK; 4 Department of Animal Histology and Embryology, University of Silesia, 9 Bankowa St., 40-007 Katowice, Poland; 5 Departamento de Biologia Aplicada à Agropecuária, Faculdade de Ciências Agrárias e Veterinárias, Univ Estadual Paulista—UNESP, Câmpus Jaboticabal, São Paulo, Brazil

**Keywords:** Bladderwort, Carnivorous plant, Floral micro-morphology, Lentibulariaceae, Osmophore, Palate, Pollination, Sect. *Iperua*, Ultrastructure

## Abstract

*Utricularia cornigera* and *Utricularia nelumbifolia* are giant, aquatic-epiphytic species of carnivorous bladderwort from southeastern Brazil that grow in the central ‘urns’ of bromeliads. Both species have large, colourful flowers. The main aim of our study is to ascertain whether the prominent floral palate of *U. cornigera* and *U. nelumbifolia* functions as an *unguentarius*—i.e. an organ that bears osmophores. Floral tissues of both species were investigated using light microscopy, scanning electron microscopy, transmission electron microscopy and histochemistry. Floral palates of *U. cornigera* and *U. nelumbifolia* provide clear visual signals for pollinating insects. In both species, the palate possesses diverse micro-morphology, comprising unicellular, conical to villiform papillae and multicellular, uniseriate, glandular trichomes that frequently display terminal branching. The most characteristic ultrastructural feature of these papillae was the presence of relatively large, polymorphic plastids (chromoplasts) containing many plastoglobuli. Similar plastids are known to occur in the fragrance-producing (osmophores) and oil-producing (elaiophores) tissues of several orchid species. Thus, these palate papillae may play a key role in providing the olfactory stimulus for the attraction of insect pollinators. Nectariferous trichomes were observed in the floral spurs of both species, and in *U. nelumbifolia*, free nectar was also recorded. The location, micro-morphology, anatomy and ultrastructure of the floral palate of the two species investigated may thus indicate that the palate functions as an unguentarius. Furthermore, the flowers of these taxa, like those of *U. reniformis*, have features consistent with bee pollination.

## Introduction

One of the largest families of carnivorous plants is Lentibulariaceae, its largest genus being *Utricularia* L., which is well known for its bladder-type traps that capture prey (Juniper et al. 1989; Adamec 2011). *Utricularia* species possess a bilabiate corolla extending posteriorly to form a floral spur. The colour of the corolla, which typically measures 5 mm–2 cm (Taylor 1989), changes to yellow and/or violet. The largest flowers for the genus occur in aquatic-epiphytic species which grow in the central ‘urns’ of bromeliads (*Utricularia cornigera* Studnička, *Utricularia nelumbifolia* Gardner and *Utricularia humboldtii* Schomb., all species of sect. *Iperua* P. Taylor), some terrestrial/lithophyte species (*Utricularia reniformis* A.St.-Hil sect. *Iperua*, *Utricularia longifolia* sect. *Phyllosperma* P. Taylor) and some epiphytic species (e.g. *Utricularia alpina* Jacq. sect. *Orchidioides* A.DC.—Taylor 1989; Guisande et al. 2007; Studnička 2009, 2011). These all have showy flowers and are often cultivated as ornamental carnivorous plants. Furthermore, some have formed the subject of embryological (Płachno and Świątek 2012), seed and seedling structure (Studnička 2009; Płachno and Świątek 2010; Menezes et al. 2014), ecological (Studnička 2011) and genetic studies (Clivati et al. 2012). With the sole exception of *U. reniformis* (Clivati et al. 2014), detailed observations of plant–pollinator interactions are lacking for these species. Pollination of the small, terrestrial species *Utricularia albocaerulea* Dalz., *Utricularia graminifolia* Vahl. (=*Utricularia purpurascens* Graham) and *Utricularia reticulata* Sm. (sect. *Oligocista* A.DC.) was reported in detail by Hobbhahn et al. (2006), who showed that they were pollinated by numerous insect pollinators, such as bees, butterflies, moths and dipterans. To date, only two species of pollinators (*Xylocopa* sp. and *Bombus* sp.) have been recorded for *U. reniformis* (Clivati et al. 2014). Recently, however, it was proposed that in the Australian species *Utricularia dunlopii*, where the nectary spur is reduced, pollinators are attracted largely by the insectiform configuration of the flower and volatilization of fragrance putatively produced by glandular trichomes (osmophores) densely distributed upon the modified floral appendages (Płachno et al. 2015).

According to Taylor (1989), the lower lip of the corolla of *Utricularia* is expanded, forming the palate. This structure is often ornamented, is pubescent or glandular, and often has diagnostic value in taxonomical studies. Moreover, the palate can easily be distinguished from the rest of the corolla owing to its distinctly different colour.

This study aims to identify the site of floral scent production and secretion in *Utricularia* sect. *Iperua*. In particular, it aims to ascertain whether the prominent palate functions as an *unguentarius*—i.e. an organ that bears scent glands or osmophores (Płachno et al. 2015). The micro-morphology of the floral spurs of both species was also investigated.

## Material and methods

**Fig. 1:**
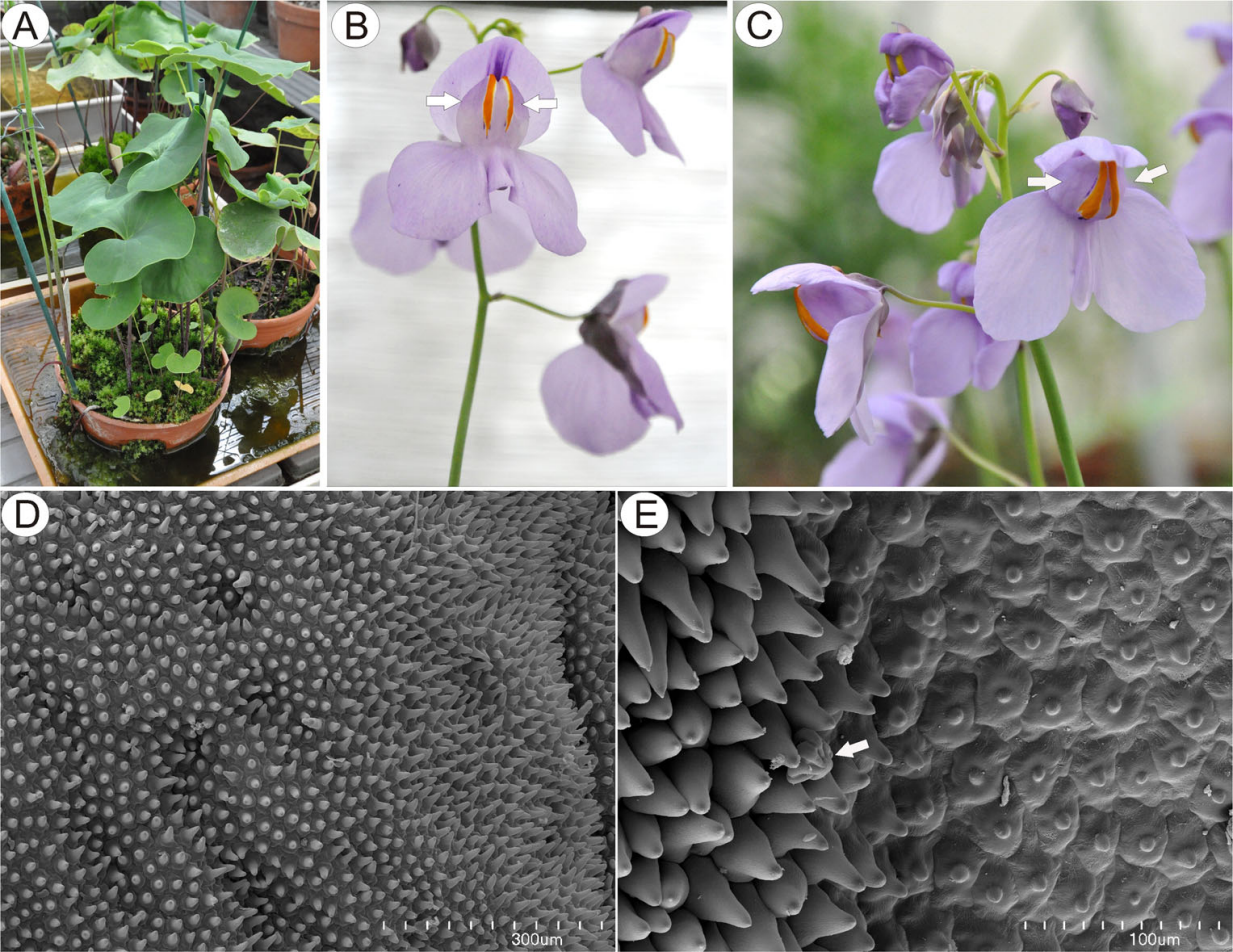
Gross and floral morphology of *Utricularia cornigera*. **a** Cultivated clone U9B at Botanic Garden Liberec. **b** Floral morphology of *Utricularia cornigera* clone U9B: palate (*arrows*) with distinct nectar guides. **c** Floral morphology of *Utricularia cornigera* clone U9: palate (*arrows*). **d** Micro-morphology of palate; *bar* = 300 μm. **e** Conical papillae (dorsal and lateral views) of palate coinciding with position of nectary guides; pollen grain (*arrow*); *bar* = 100 μm

Species used in this study include *U. cornigera* Studnička clone U9B (which was used as the holotype, Studnička 2009) and clone U9 obtained from Botanická zahrada Liberec, Czech Republic (Fig. 1a–c) and *U. nelumbifolia* Gardner obtained from the living collections of Jagiellonian University Botanical Garden in Kraków. Some additional material was provided by Botanická zahrada Liberec, Czech Republic (Fig. 8a, b).

### Floral structure and histochemistry

The distribution of secretory glandular trichomes and unicellular papillae was determined by examining entire flowers using a Nikon SZ100 stereoscopic microscope. We interpret the term ‘palate’ in *Utricularia* as the inflated base of the lower lip of the corolla, which differs both morphologically and in terms of colour from the remaining part of the perianth.

Floral parts bearing papillae and glandular trichomes, namely the palate and spur, were examined using light microscopy (LM), scanning electron microscopy (SEM) and transmission electron microscopy (TEM), as follows: Firstly, the epidermis of the floral palate was examined during anthesis, and pieces of floral tissue were excised and fixed in 2.5 % (*v*/*v*) glutaraldehyde 2.5 % (*v*/*v*) formaldehyde in 0.05 M sodium cacodylate buffer (pH 7.0) for 2 h at 4 °C, washed three times in 0.05 sodium cacodylate buffer pH 7 and post-fixed in 1 % (*w*/*v*) osmium tetroxide solution for 1.5 h at 0 °C. Dehydration using a graded ethanol series and infiltration and embedding using an epoxy embedding medium kit (Fluka) followed. Following polymerization at 60 °C, sections were cut at 70 nm for TEM using a Leica ultracut UCT ultramicrotome, stained with uranyl acetate and lead citrate (Reynolds 1963), and examined using a Hitachi H500 transmission electron microscope at an accelerating voltage of 75 kV in the Faculty of Biology and Environmental Protection, University of Silesia in Katowice and a Jeol JEM 100 SX; JEOL, Tokyo, Japan, at 80 kV in the Department of Cell Biology and Imaging, Institute of Zoology, Jagiellonian University in Kraków.

Semi-thin sections (0.9–1.0 μm thick) were prepared for light microscopy (LM) and stained for general histology using aqueous methylene blue/azure II (MB/AII) for 1–2 min (Humphrey and Pittman 1974) and examined with an Olympus BX60 light microscope. The periodic acid-Schiff (PAS) reaction was also used to reveal the presence of insoluble polysaccharides, and Sudan Black B was used to detect the presence of lipids (Jensen 1962). Staining for total proteins was achieved using Coomassie brilliant blue R250 or Ponceau 2R (Fisher 1968; Ruzin 1999). Material was also tested for lipids, starch and mucilage using a saturated ethanolic solution of Sudan III, aqueous IKI (iodine-potassium iodide) solution and ruthenium red solution, respectively (Ruzin 1999).

A Nikon Eclipse E200 camera and an Olympus BX60 microscope were used for general photography and micrometry/photomicrography, respectively.

For SEM, the representative floral parts were dehydrated and subjected to critical point drying using liquid CO_2_. They were then sputter-coated with gold and examined at an accelerating voltage of 20 kV using a Hitachi S-4700 scanning electron microscope (Hitachi, Tokyo, Japan) based at the Scanning Microscopy Laboratory of the Department of Biological and Geological Sciences, Jagiellonian University in Kraków.

## Results

### *U. cornigera* Studnička

#### Floral structure

**Fig. 2:**
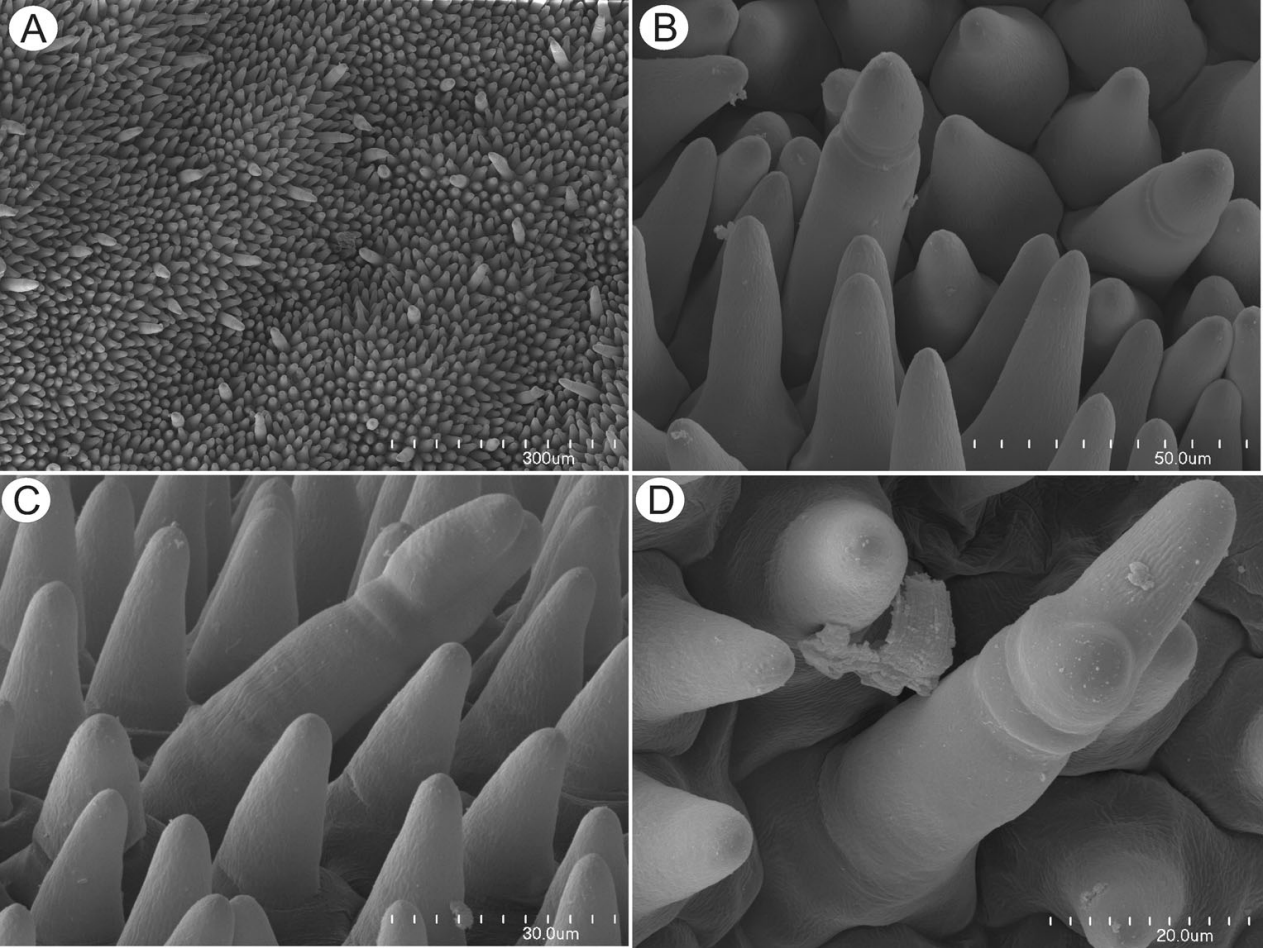
Micro-morphology of *Utricularia cornigera* palate. **a** Papillae and glandular trichomes; *bar* = 300 μm. **b** Glandular trichomes with acorn-shaped head; *bar* = 50 μm. **c** Glandular trichome with bicellular head; head cells are of similar size; *bar* = 30 μm. **d** Glandular trichome with bicellular head; head cells differ in size; *bar* = 20 μm

**Fig. 3:**
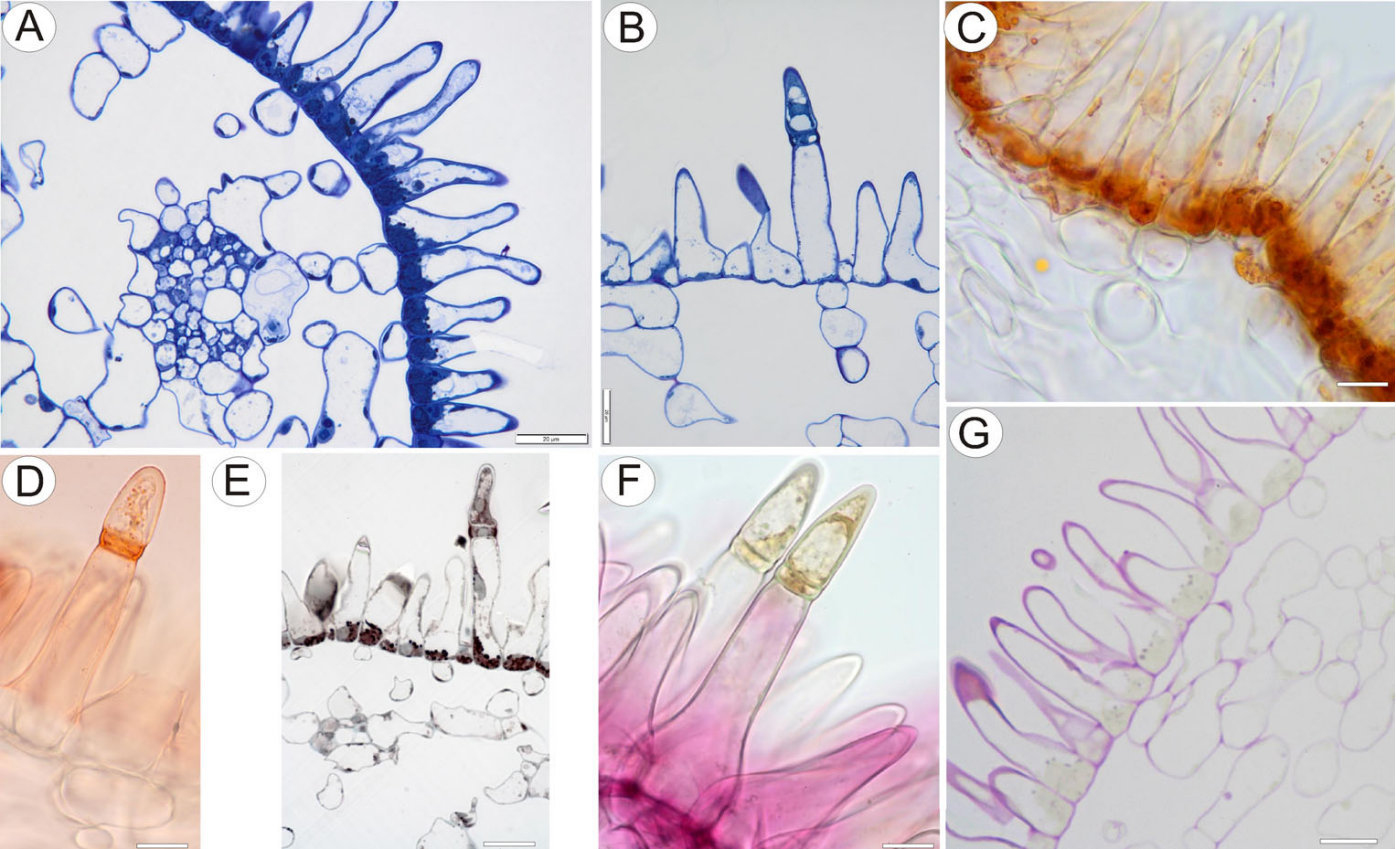
Anatomy and histochemistry of palate of *Utricularia cornigera*. **a** Section of palate with papillose epidermis and subepidermal parenchyma showing large intercellular spaces (MB/AII); *bar* = 20 μm. **b** Glandular trichome amongst epidermal papillae. Note that head cells of trichome stain intensely with MB/AII; *bar* = 20 μm. **c** Numerous chromoplasts in the cytoplasm of the basal part of papillae and lipid droplets stained with Sudan III; *bar* = 20 μm. **d**, **e** Lateral cell walls of the central cell of glandular trichome stained with Sudan III and Sudan Black B, respectively. In **e**, chromoplasts are also stained black; *bars* = 15 and 26 μm, respectively. **f** Epidermal papillae and glandular trichomes stained with ruthenium red. Penetration of head cells by stain is slower than for other cells; *bar* = 13 μm. **g** The PAS reaction did not indicate the presence of starch in palate cells; *bar* = 25 μm

Flowers were large and remained closed. The lower lip of the corolla was expanded to form a wide platform, the palate (Fig. 1b, c). The inflated palate was relatively massive, blue-violet with two vertical, prominent orange marks with white margins (Fig. 1b, c), which function as nectar guides. The adaxial epidermal surface of the palate was single layered. Although papillae occurred over the entire surface of the palate, many of those located on the nectar guides were more distinctly conical to villiform (Figs. 1d, e, 2a–c, and 3a, c, g). The surface of these papillae was almost smooth, and the overlying cuticle lacked cracks. SEM observations did not reveal the presence of secretion on the surface of papillae; however, some debris was present. Scattered between the papillae were stalked, multicellular, uniseriate, glandular trichomes (Figs. 2a–d and 3b, d–f) of two types. Both possessed a long stalk (mean length = 63.9 μm, *n* = 20), a shorter central cell (mean length = 5.2 μm, *n* = 20) and a head (mean length = 26.0 μm, *n* = 20). In the first type, the head was unicellular, swollen and acorn-shaped, whereas in the other, it was branched and bicellular (Fig. 2b–d). Such hairs formed approx. 2.5 % of palate epidermal cells. The subepidermal mesophyll was parenchymatous and consisted of highly vacuolate, non-secretory cells (Fig. 3a, b, e, g).

**Fig. 4:**
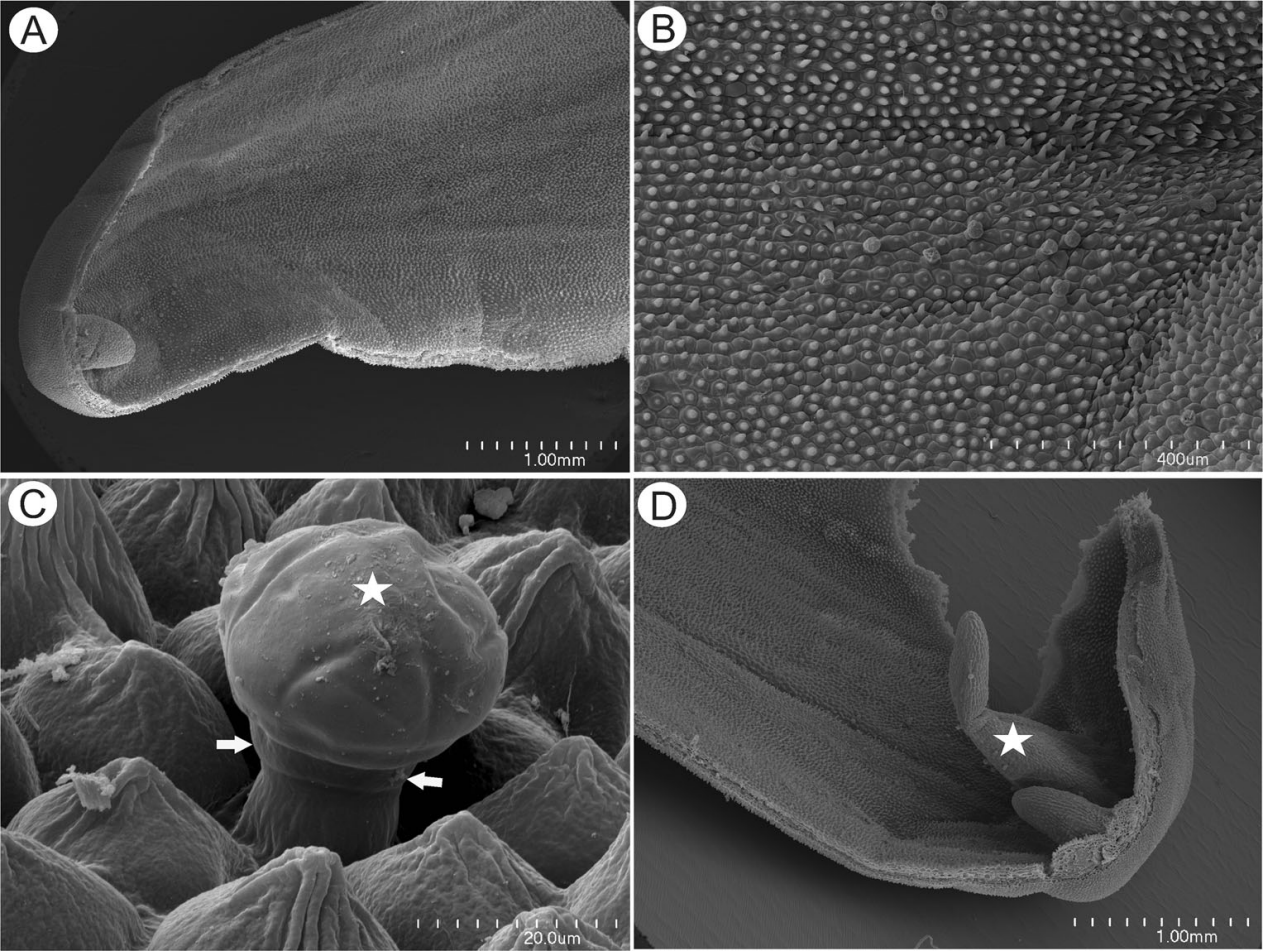
Micro-morphology of *Utricularia cornigera* nectary spur. **a** Apical part of the spur; *bar* = 1 mm. **b** Inner adaxial surface of nectary spur with glandular trichomes and small, conical papillae; *bar* = 400 μm. **c** Glandular trichome amongst conical papillae showing cuticular striations, the central cell (*arrows*) and multicellular head (*star*); *bar* = 20 μm. **d** Bud-like structure (*star*) within apex of the nectary spur; *bar* = 1 mm

**Fig. 5:**
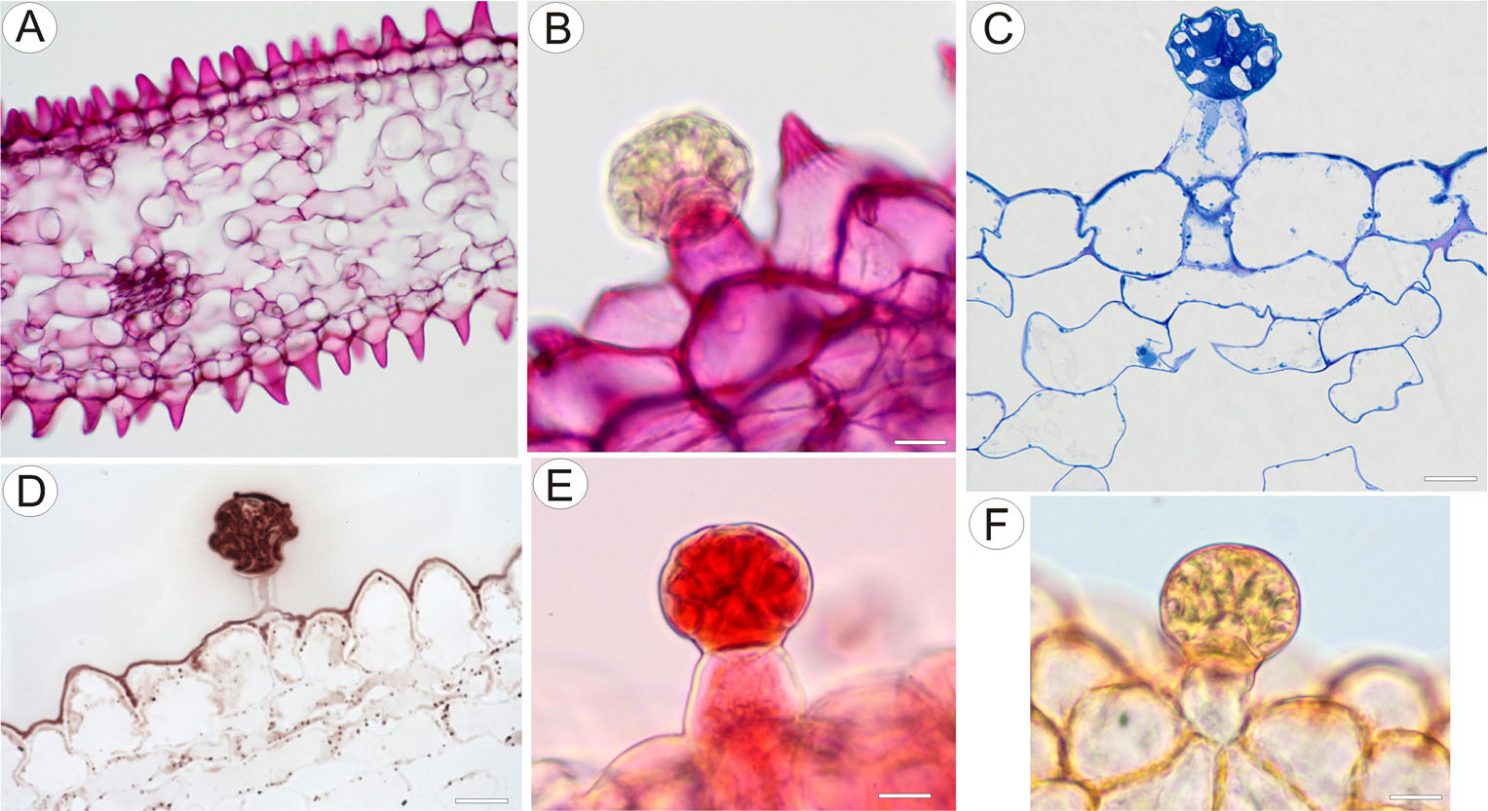
Anatomy and histochemistry of the spur of *Utricularia cornigera*. **a** Section of the proximal part of the spur stained with ruthenium red; *bar* = 40 μm. **b** Glandular trichome and conical papillae stained with ruthenium red. Note that staining of the head cells is slower; *bar* = 13 μm. **c** Glandular trichome stained with MB/AII; *bar* = 9 μm. **d** Cytoplasm of the head cells intensely stained with Ponceau 2R; *bar* = 13 μm. **e**, **f** Staining of glandular trichomes with Sudan Black B and Sudan III, respectively. Note that in **e**, the uniform cuticle and plastids have stained black with this reagent; *bars* = 20 and 13 μm, respectively

The prominent, cylindrical floral spur projected parallel to and between the lobes of the lower lip of the corolla. Whereas the outer epidermis of the spur was predominantly papillose, the inner comprised conical papillae proximally but was glabrous with flattened epidermal cells or with globose papillae distally (Figs. 4a–d and 5a–f). The cuticle of the conical papillae was striate (Figs. 4c and 5d). Capitate, glandular trichomes occurred between these cells (Figs. 4b, c and 5b–f), consisting of a unicellular stalk (mean length = 30.7 μm), a short, central cell (mean length = 15.2 μm) and a head comprising 8–10 cells (mean length = 16.7 μm). The parenchymatous cells of the spur wall were irregularly shaped, with prominent intercellular spaces (Fig. 5a, c). Swollen, bud-like structures, possibly adventitious buds or vestiges of the apices of the individual, fused perianth segments that form the nectary spur, were present at the spur apex (Fig. 4d).

#### Histochemistry

Testing with IKI and PAS (Fig. 3g) did not reveal the presence of starch in epidermal and parenchyma cells of the palate. However, testing hand-sectioned material with Sudan III (Fig. 3c, d), and semi-thin sections with SBB (Fig. 3e), indicated the presence of lipids in the plastids of papillose epidermal cells and in trichomes, and several lipid droplets were observed in the cytoplasm (Fig. 3c, e). The lateral cell walls of the short central cell stained selectively with both Sudan III and Sudan black B. Ruthenium red (Fig. 3f) and Ponceau 2R neither detected the presence of mucilage nor storage proteins, respectively. The dense cytoplasm of both the central cells and head cells of trichomes stained strongly with MB/AII, whereas the stalk cell was highly vacuolate, containing only parietal cytoplasm (Fig. 3b).

Staining of the floral spur with ruthenium red did not indicate the presence of mucilage in the head cells of secretory trichomes and epidermal papillae (Fig. 5a, b). It is possible that the thick impermeable cuticle of head cells, coupled with its hydrophobic nature, inhibited penetration by aqueous stains. The cytoplasm of the head cells stained intensely with MB/AII and with Ponceau 2R indicating an elevated protein content. Treatment with Sudan stains did not indicate the presence of lipids in the cytoplasm of cells lining the spur, nor did these reagents stain the cell walls of glandular trichomes. However, they selectively stained the uniform cuticle overlying the outer walls of cells enclosing the lumen (Fig. 5e, f).

#### Ultrastructural studies

**Fig. 6:**
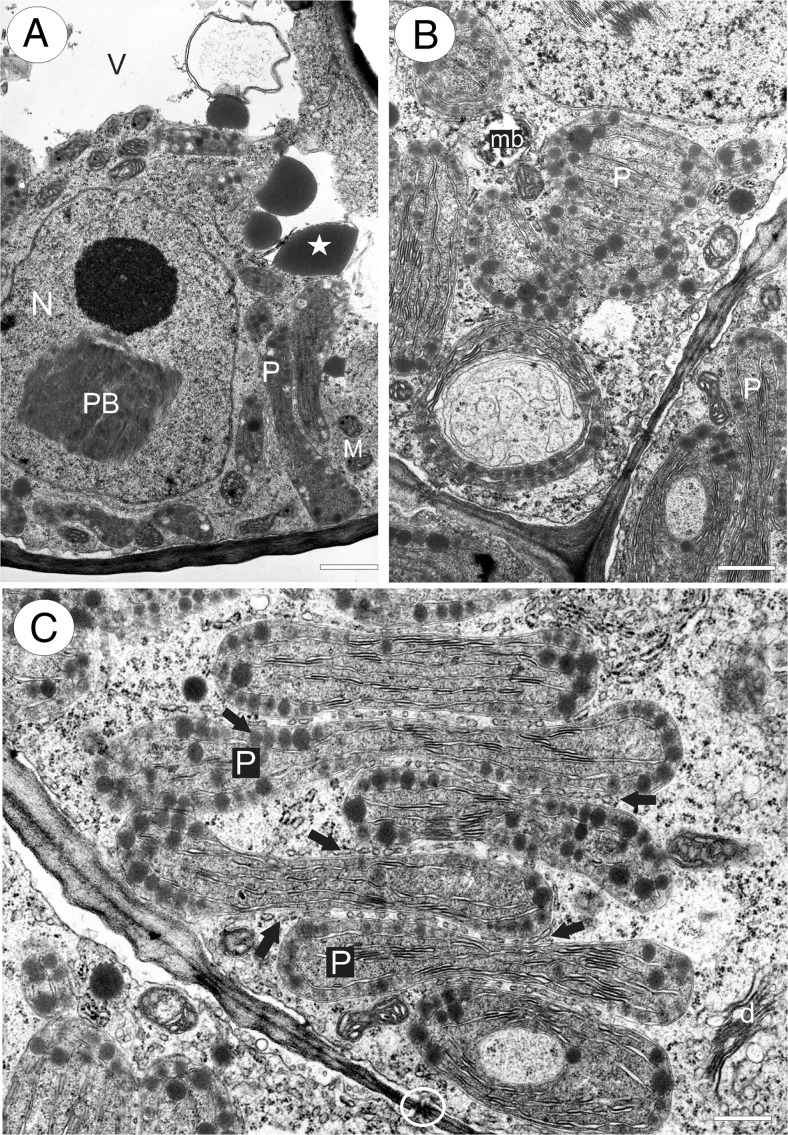
Ultrastructure of *Utricularia cornigera* palate papillae. **a** General ultrastructure of the basal part of papillae; *bar* = 1 μm. **b**, **c** Polymorphic plastids in papillae. Note the numerous osmiophilic plastoglobuli within plastids and that plastids are closely associated with the endoplasmic reticulum (*arrows*); *bars* = 0.8 and 0.6 μm; *d* dictyosome, *M* mitochondria, *mb* microbody, *n* nucleus, *PB* intranuclear paracrystalline body, *V* vacuole; *circle* plasmodesmata, *star* osmiophilic body

**Fig. 7:**
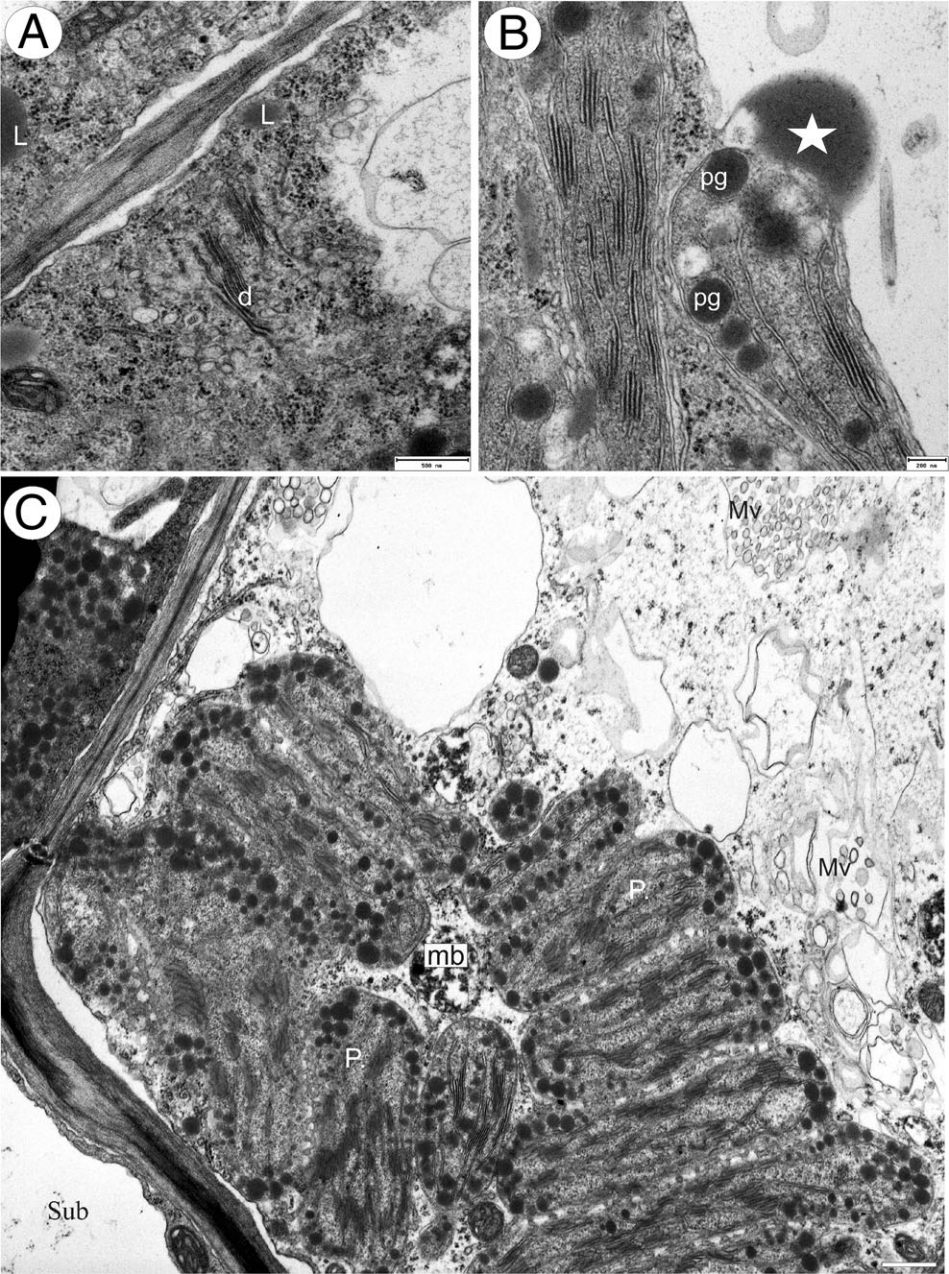
Ultrastructure of *Utricularia cornigera* palate papillae. **a** Electron micrograph showing dictyosomes (*d*) with numerous small vesicles and cytoplasmic lipid bodies (*L*); *bar* = 500 nm. **b** Plastids containing numerous, large plastoglobuli. Note the osmiophilic body (*star*) associated with plastid; *bar* = 200 nm. **c** General ultrastructure of the basal part of papilla. Note the multi-vesicular bodies (*Mv*), microbodies (*mb*), plastids (*P*), and part of subepidermal cell (*Sub*); *bar* = 0.9 μm

The palate papillae were nucleate and contained electron-dense cytoplasm. The nucleus was located at the base of the cell, whereas a large vacuole was often present in the papilla projection. Intranuclear, paracrystalline protein inclusions were occasionally present (Fig. 6a). The cytoplasm in the basal part of the papilla was particularly rich in organelles, such as plastids and mitochondria (Fig. 6a–c). The most remarkable feature of these cells was the large, oval or polymorphic chromoplasts (often with cup-shaped and irregular profiles). These plastids had well-developed internal membranes with dilated cisternae and also contained numerous, large, lipid globules or plastoglobuli (Fig. 6b, c). Starch grains were generally absent. Endoplasmic reticulum often occurred in close proximity to the plastids. The cytoplasm contained abundant rough endoplasmic reticulum (RER), and dictyosomes, though present, were not common. Many small vesicles occurred close to the dictyosomes (Fig. 7a). Lipid bodies, some of them occurring close to the plastids, and microbodies were also frequent (Figs. 6b and 7c). Mitochondria were elongate with numerous, well-developed cristae. Vacuoles contained large osmiophilic bodies (Fig. 6a), and plastids seemingly contributed towards their formation (Fig. 7b). Similar osmiophilic bodies also occurred in the cytoplasm. Some vacuoles also contained large, membranous, myelin-like intravacuolar bodies or multi-vesicular bodies (Fig. 7c). Plasmodesmata present in primary pit-fields maintained cytoplasmic continuity between contiguous epidermal cells (Fig. 7c). In contrast, subepidermal cells were highly vacuolate and lacked plastids of the kind found in papillae.

### *U. nelumbifolia* Gardner

#### Floral structure

**Fig. 8:**
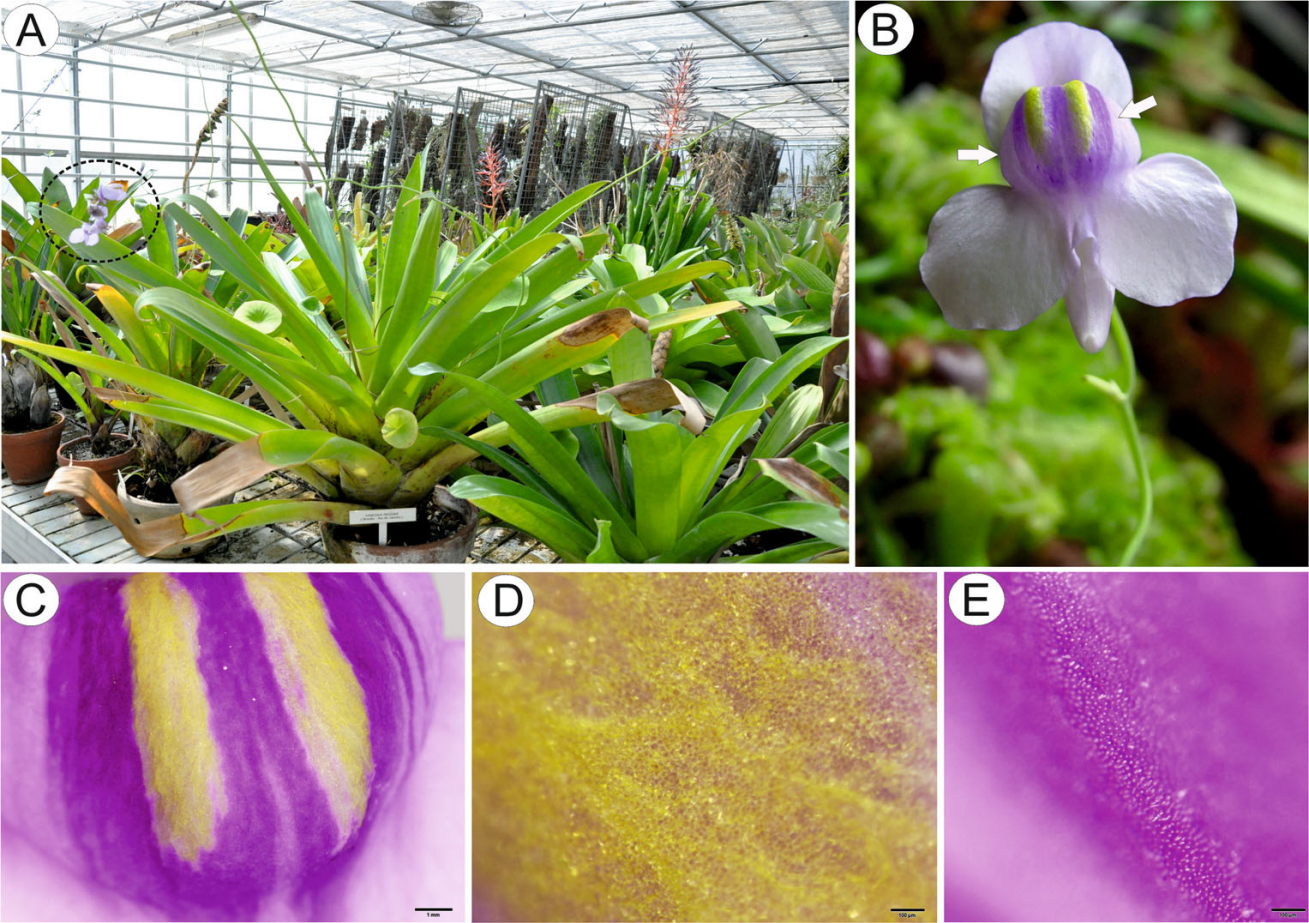
Gross and floral morphology of *Utricularia nelumbifolia*. **a** Cultivated plant *Utricularia nelumbifolia* in the tank of *Vriesea regina* (Vell.) Beer in Botanic Garden Liberec; flowers (*circle*). **b** Detail of entire flower showing palate (*arrows*). **c** Morphology of the palate. Note the distinct nectar guides; *bar* = 1 mm. **d** Micro-morphology of palate showing the surface of nectar guide; *bar* = 100 μm. **e** Micro-morphology of the palate; note the glistening papillae and trichomes; *bar* = 100 μm

**Fig. 9:**
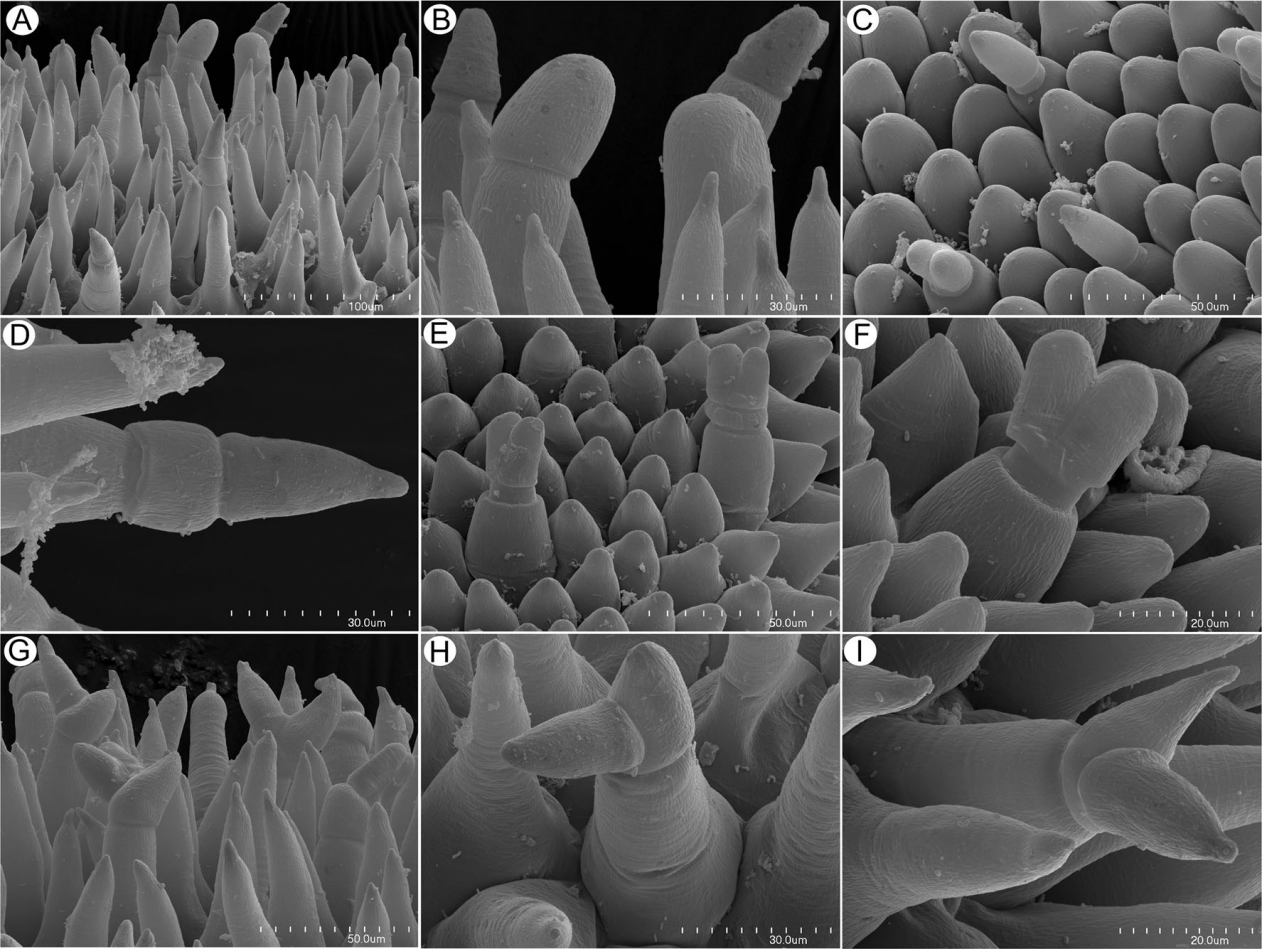
Detail of palate *Utricularia nelumbifolia* showing diversity of glandular trichomes and conical papillae. **a** Papillae and various types of trichome; *bar* = 100 μm. **b**, **c** Trichomes with unicellular acorn-shaped head. Note the variation in size of trichome heads; *bars* = 30 and 50 μm, respectively. **d** Trichome with unicellular, pointed head. Note the distinct central cell; *bar* = 30 μm. **e**, **f** Trichomes with bicellular head and squat stalk cell; *bars* = 50 and 20 μm, respectively. **g**–**i** Trichomes with branched, multicellular heads. Again, note the range in the size of head cells; *bars* = 50, 30, and 20 μm, respectively

**Fig. 10:**
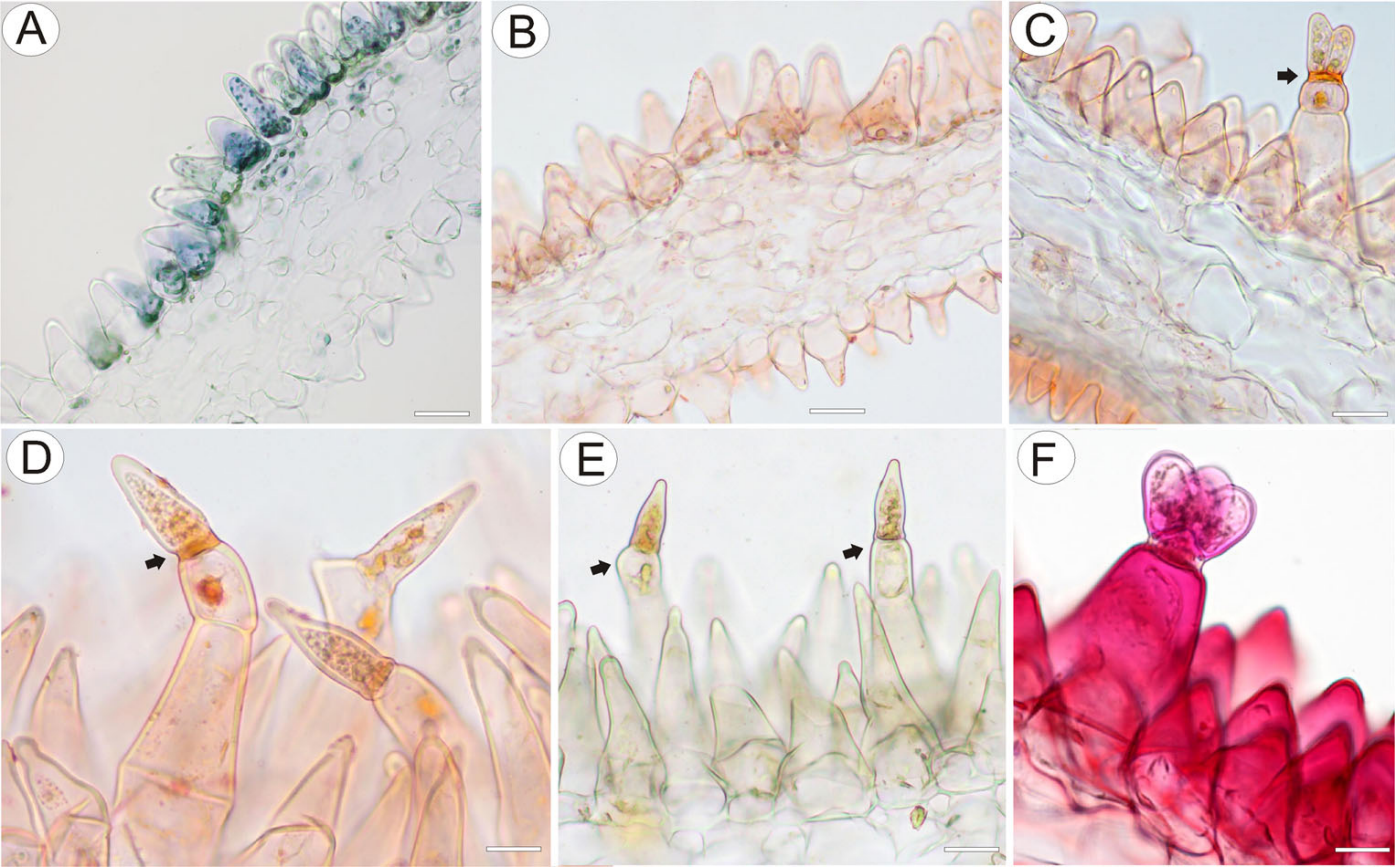
Anatomy and histochemistry of palate of *Utricularia nelumbifolia*. **a** Cytoplasm with plastids in papillose cells stained with Coomassie brilliant blue; *bar* = 45 μm. **b**–**d** Epidermal papillae and trichomes stained with Sudan III. **b** Papillae with chromoplasts and lipid droplets; *bar* = 35 μm. **c** Glandular trichome composed of a bicellular stalk, a short central cell, and a bicellular head; *bar* = 20 μm. **d** Glandular trichomes with pointed terminal cells; *bar* = 15 μm. In **c** and **d**, note the selectively stained lateral cell walls of central cell (*arrows*) that may function as a hydrophobic barrier. **e** Testing with IKI did not indicate the presence of starch in papillae and glandular trichomes; *bar* = 22 μm. **f** Epidermal conical papillae and glandular trichome stained with ruthenium red. Note the unstained, impermeable lateral cell walls of the central cell (*arrows*); *bar* = 12 μm

Again, flowers were large and remained closed. The lower lip of the corolla was expanded to form a wide platform, the palate (Fig. 8b, c). The inflated palate was relatively massive, dark violet at its centre, with two vertical, prominent yellow marks with white margins (Fig. 8c), which function as nectar guides. The adaxial epidermal surface consisted of conical papillae (Figs. 8d, e and 9a–i). The surface of these papillae was smooth, the overlying cuticle lacking cracks. Debris and bacteria were present on the surface of the papillae, and scattered amongst these papillae were multicellular, uniseriate trichomes (measuring 95.2–156.5 μm, *n* = 20) (Fig. 9a). The stalk was composed of one to three cells (Figs. 9a–i and 10c, d, *n* = 20) and the head was attached to a short, central cell (measuring 3.5–6.5 μm in length, *n* = 20). Whereas some trichomes consisted of one to three cells, including a swollen, unicellular, acorn-shaped head (Figs. 9b, c and 10c, f), others had a pointed terminal cell (Figs. 9d–f and 10d) or a branched multicellular head consisting of one to three cells (Fig. 9g, h). Micro-droplets of secretion were observed on the surface of trichomes (Fig. 9h, i). Such trichomes formed approx. 4.5–7.0 % of palate epidermal cells.

**Fig. 11:**
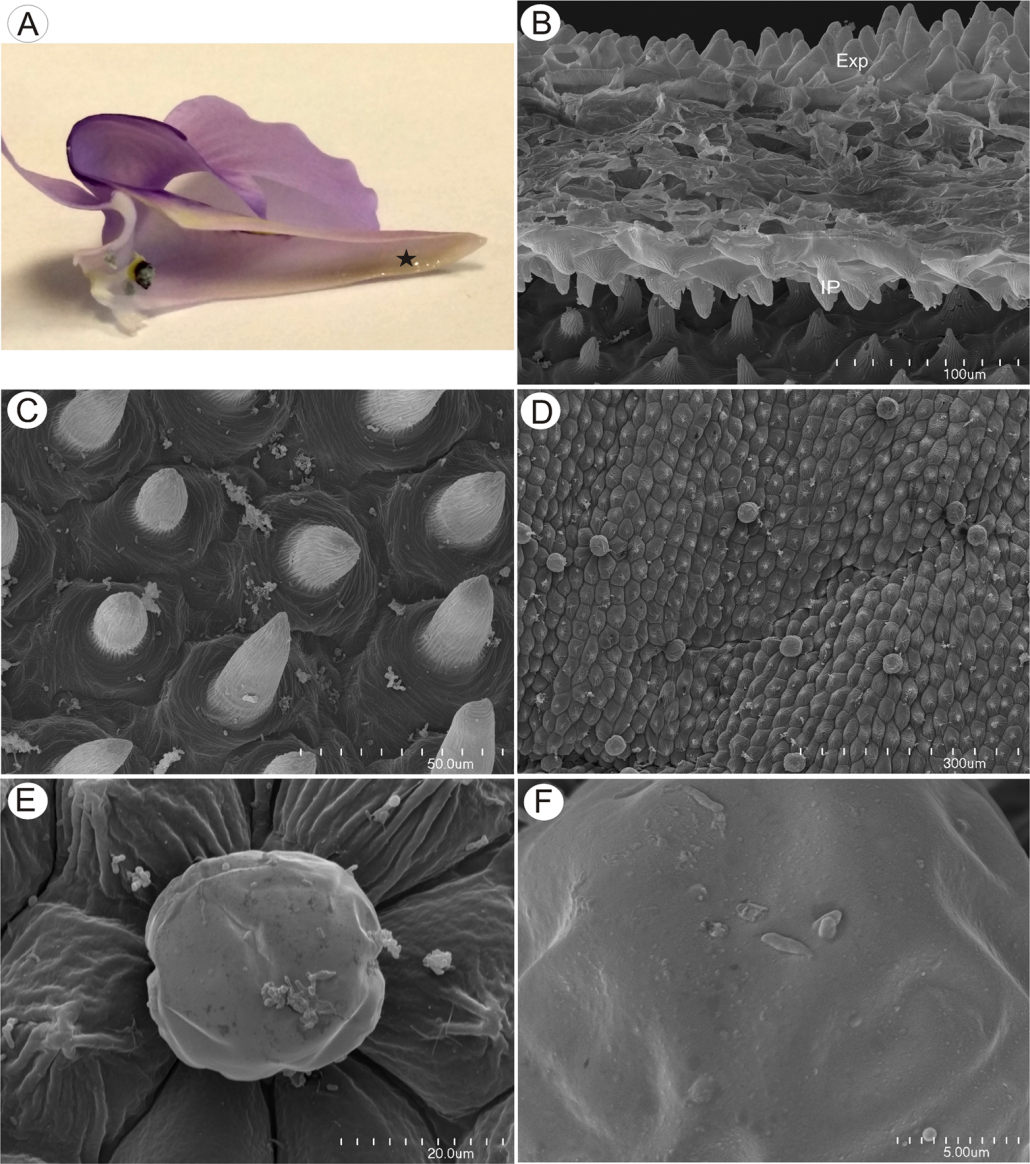
Nectary spur structure and micro-morphology of *Utricularia nelumbifolia*. **a** Section through flower. Note the presence of nectar (*star*) within the spur. **b** Adaxial wall of spur showing papillose external surface (*ExP*) and papillae with cuticular striations enclosing the lumen (*IP*); *bar* = 100 μm. **c** Small, conical papillae with cuticular striations on the internal adaxial surface of the spur; *bar* = 50 μm. **d** Internal, adaxial surface of spur with conical papillae and nectar-secreting glandular trichomes; *bar* = 300 μm. **e** Nectar-secreting glandular trichome; *bar* = 20 μm. **f** Surface of head cells of glandular trichome with micro-droplets of secretion; *bar* = 5 μm

**Fig. 12:**
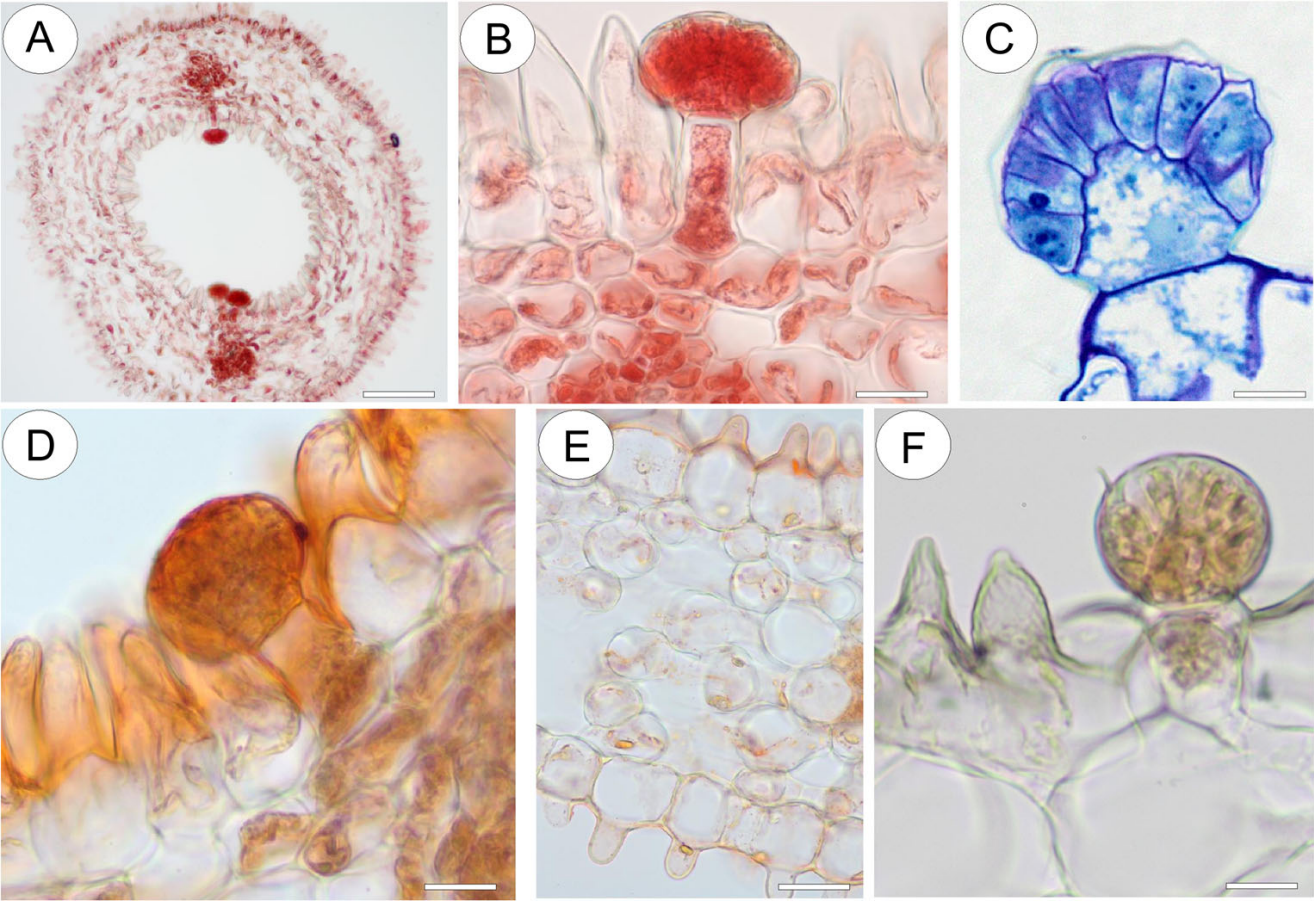
Anatomy and histochemistry of spur of *Utricularia nelumbifolia*. **a** Apical part of spur stained with Ponceau 2R. Glandular trichome distribution coincides with the position of vascular bundles; *bar* = 150 μm. **b** Head cells of glandular trichome stained intensely with Ponceau 2R; *bar* = 20 μm. **c** Detail of the head of glandular trichome following staining with MB/AII. Note the dense cytoplasm of the secretory head cells and that the cuticle has become detached from the outer cell walls; *bar* = 14 μm. **d** Cuticle overlying the glandular trichome stained uniformly with Sudan III; *bar* = 20 μm. **e** Epidermal and parenchyma cells of the spur contain small lipid droplets; *bar* = 30 μm. **f** Testing with IKI did not indicate the presence of starch in the conical cells of the epidermis nor the glandular trichomes; *bar* = 20 μm

The subulate floral spur was prominent and projected parallel to and between the lobes of the lower lip of the corolla (Fig. 8b). The apical part of the nectary spur contained nectar (Fig. 11a). Both external and internal epidermal surfaces of the spur were papillose. Conical to villiform papillae predominated in the latter (Fig. 11b–d), and these had a striate cuticle (Fig. 11c). Within the spur, enclosing the lumen, were multicellular, uniseriate, capitate, shortly stalked, glandular trichomes (Figs. 11d, e and 12a–d, f). These were distributed predominantly along two tracts coinciding with the main vascular bundles (Fig. 12a) in the apical part of the nectary spur, and there is evidence that the cuticle overlying the secretory multicellular head becomes distended in response to the subcuticular accumulation of nectar (Fig. 12c). Micro-droplets of secretions were also observed on the head cells of these capitate trichomes (Fig. 11f).

#### Histochemistry

Treatment of the epidermal cells of the palate with Coomassie Brilliant Blue and Ponceau 2R did not reveal the presence of cytoplasmic protein bodies (Fig. 10a). Treatment with Sudan III, however, revealed numerous lipid droplets in the cytoplasm of both papillae and trichomes (Fig. 10b–d), whereas treatment with IKI and ruthenium red, respectively, failed to demonstrate the presence of starch and mucilage (Fig. 10e and f, respectively). With the exception of individual grains in parenchyma cells of the vascular bundle sheath, starch was not detected in cells of the palate (Fig. 10e). Ruthenium red stained only cell walls (Fig. 10f).

The distribution of these various ergastic substances, as revealed by histochemical analysis of both the palate and spur, closely resembled that obtained for *U. cornigera* (Fig. 12a–f).

#### Ultrastructural studies

In *U. nelumbifolia*, the ultrastructure of the palate papillae resembled that of *U. cornigera*. Again, the most outstanding ultrastructural feature was the presence of large, polymorphic chromoplasts containing numerous, large lipid globules or plastoglobuli (not shown).

## Discussion

### Floral palate

The contrasting colouration of the various parts of the floral palate (blue-purple or dark purple backgrounds and orange or yellow nectar guides) hints strongly at the importance of the palate in the attraction of potential insect pollinators, and it is probable that such contrasting colours can be perceived from afar and guide the pollinator both to the reproductive parts of the flower and to the nectar located in the floral spur. The tall inflorescences of both species (up to 1.2 m in *U. nelumbifolia*; Taylor 1989) may play an important role in presenting the flowers to full advantage above the robust, strap-like leaves of bromeliads, especially since they sway in the wind (as in *U. reniformis*), thus further drawing the attention of pollinators to the flowers.

Indeed, both the colour and shape of the palate of *U. cornigera* and *U. nelumbifolia* also resemble those of *U. reniformis*, a species which, according to Clivati et al. (2014), based on testing tissues with aqueous neutral red solution, lacks osmophores (although this may simply be due to the hydrophobic nature of the epidermal surface). Recently, we proposed that the floral glandular hairs of *U. dunlopii* P. Taylor, a member of sect. *Pleiochasia*, having a small palate and two and three, long, erect, filiform appendages arising from the upper and lower lips of the corolla, respectively, may function as osmophores (Płachno et al. 2015). By contrast, other members of the same section have a well-developed palate (e.g. *Utricularia paulinae* Lowrie, *Utricularia uniflora* R.Br. and *Utricularia dichotoma* Labill.) that may also function as an unguentarius, especially since it is papillose in all these species, and in *U. uniflora* and *U. paulinae*, it also bears glandular trichomes (Płachno et al. 2015).

Until recently, the term ‘osmophore’ was used to describe both the fragrance-secreting cell and the structure that bears it (Vogel 1990). In order to avoid confusion, we proposed that the latter, in the future, should be referred to as the unguentarius (Płachno et al. 2015). Often, in orchids such as *Ophrys* L., the unguentarius is the labellum and the osmophores it bears are dome-shaped or lenticular (Ascensao et al. 2005; Bradshaw et al. 2010; Francisco and Ascensão 2013). In other orchids, the unguentarius may be a modified, antenniform dorsal sepal (e.g. *Restrepia* Kunth—Pridgeon and Stern 1983) or the lateral and/or dorsal sepals (e.g. *Scaphosepalum* Pfitzer—Pridgeon and Stern 1985), or even projections of the labellum (e.g. *Chloraea membranacea* Lindl.—Sanguinetti et al. 2012). These all bear specialized osmophores. Often, however, osmophores are represented merely by conical papillae, such as those that occur on the labella of the species of *Cymbidium* Sw. (Stpiczyńska 1993; Davies et al. 2006) and *Gymnadenia conopsea* (L.) R.Br. (Stpiczyńska 2001).

The palate surface of both *U. cornigera and U. nelumbifolia* consisted primarily of conical to villiform, unicellular papillae. These cells are ubiquitous amongst angiosperms but, being the most commonly encountered type of floral epidermal cell (Kay et al. 1981), are of little value in establishing taxonomic relationships. At first, their relatively unspecialized form and ubiquity would suggest that they are not involved in specialized physiological activities such as fragrance production. However, as has already been stated, in certain fragrant species where the perianth consists solely of such cells, it has been established that these cells must be the source of the fragrance and thus function as osmophores (Stpiczyńska 1993, 2001). Their surprisingly dense cytoplasm, possessing an organelle complement consistent with secretory activity, together with the selective uptake of Sudan stains by these papillae in *U. cornigera* and *U. nelumbifolia*, may indicate that they too function as osmophores.

Of greater significance, perhaps, as potential osmophores (based on comparisons of their micro-morphology with the osmophores of other unrelated taxa) are the multicellular, uniseriate, glandular trichomes that form only 2.5–7.5 % of the epidermal cells comprising the floral palate of *U. cornigera* and *U. nelumbifolia*. Palate trichomes with conical apical cells similar to those of *U. cornigera* and *U. nelumbifolia* also occur in sect. *Utricularia* (e.g. *Utricularia aurea* Lour. = *Utricularia flexuosa* Vahl and *Utricularia inflexa* Forssk. = *Utricularia stellaris* var. *inflexa* (Forssk.) C.B.Clarke) on the adaxial surface of the corolla in the throat region (Khan 1954; Farooq 1963). General staining of the palate tissue of *U. cornigera* and *U. nelumbifolia* with MB/AII revealed that the terminal cells of these trichomes also possess dense, organelle-rich cytoplasm and are thus probably involved in secretion. Alcoholic stain solutions such as Sudan III penetrate the head cells quickly and, in this particular case, reveal that they contain lipid bodies and are involved in the metabolism of lipids (or related materials including oils, terpenoids, fragrances and resins), whereas aqueous stain solutions penetrate slowly, possibly due to the hydrophobic nature of the cell wall. Thus, it would appear that either there are two epidermal structures involved with fragrance production in these species (papillae and trichomes) or that one of these structures is involved in the secretion of other lipid-related compounds of unknown function.

Transmission electron microscopy demonstrated the presence of an organelle complement characteristic of secretory cells in these epidermal papillae, including a relatively large nucleus, abundant RER profiles and free ribosomes, occasional dictyosomes and mitochondria with numerous and well-developed cristae. One remarkable and noteworthy characteristic was the presence of numerous, oval to irregularly shaped chromoplasts, each containing well-developed internal lamellae with dilated cisternae and numerous oil bodies or plastoglobuli of various sizes, which Lange and Turner (2013) consider to be a feature of cells involved in the synthesis of terpenoids or fragrance precursors. Indeed, such plastids have been reported from the fragrance-producing tissues of several orchids including *Anacamptis pyramidalis* (L.) Rich. (Kowalkowska et al. 2012) and *Gongora bufonia* Lindl. (Adachi et al. 2015). Similar oil bodies also occur scattered throughout the cytoplasm of *U. cornigera* and *U. nelumbifolia* and may accumulate within multi-vesicular bodies or components of the vacuome, including small vesicles that accumulate next to the plasmalemma. It would thus appear that oils and lipids synthesized in plastids are discharged into the cytoplasm and undergo vesicle-mediated transport to the plasmalemma, or become associated with the ER, or are stored within vacuoles. Therefore, in many ways, these cells resemble the osmophore cells described for a range of non-related taxa, in particular, those of Orchidaceae (Pridgeon and Stern 1983, 1985; Stern et al. 1987; Stpiczyńska 1993, 2001; Sanguinetti et al. 2012).

In many orchids, lipids (including precursors of fragrance production) may traverse the outer cell wall as moieties of low molecular weight (Davies et al. 2003) and pass along micro-channels in the cuticle (e.g. Sanguinetti et al. 2012) before accumulating on (floral food reward oils) or evaporating from (fragrances) the surface of the epidermis. However, unlike the less volatile, lipid-rich food rewards of greater molecular weight produced by a number of orchids (e.g. by the labella of certain orchids—Davies et al. 2003; and in particular, the elaiophores of Oncidiinae orchids; Davies et al. 2014 and references therein), these fragrances leave little residue—a further feature of the palate epidermis of the investigated species.

Generally, ‘osmophores’ (strictly, the unguentarius) consist of an epidermis and subepidermal (subsecretory parenchyma) layer(s) (e.g. Vogel 1990; Curry et al. 1991; Stpiczyńska 2003; Płachno et al. 2010; Antoń et al. 2012), the latter usually containing numerous starch grains and contributing to the secretory process by providing energy formed by hydrolysis of this polysaccharide. However, in the palate of *U. cornigera* and *U. nelumbifolia* (based on ultrastructure and histochemistry), and the osmophores of the orchid *Grobya amherstiae* Lindl. (Pansarin et al. 2009), only the epidermis appears to be physiologically very active. Starch also commonly occurs in conjunction with lipid droplets, in osmophore cells (Vogel 1990), although this is not always the case, e.g. starchless plastids occur in the osmophores of *G. conopsea* (Stpiczyńska 2001).

Based on the evidence that these trichomes and papillae are largely restricted in distribution to the floral palate, are morphologically specialized (at least in the case of trichomes) and contain an organelle complement consistent with high rates of metabolism and lipid synthesis, we must conclude that these epidermal structures possess many of the characters of osmophores, and therefore, the palate probably functions as an unguentarius.

That said, it must also be acknowledged that the flowers that form the subjects of this paper lacked perceptible fragrance. However, it is now known that many species produce relatively strong and effective fragrances that cannot be perceived by humans (Proctor et al. 1996; Dudareva and Pichersky 2006). Nevertheless, at this stage, we cannot categorically state that the palate papillae and trichomes of *U. cornigera* and *U. nelumbifolia* function exclusively in fragrance production, since they may also secrete other lipid-rich compounds that help conserve or repel water, or may possibly deter herbivores or may simply provide tactile cues for potential insect pollinators that alight on the corolla. Only when it is possible to extract and analyse volatiles produced by minute pieces of tissue consisting solely of one type of cell, will it be possible to state categorically which components of the palate epidermis function as osmophores.

### Nectary spur

Generally, co-evolution has resulted in the mutual development of the nectary spur and insect proboscis relative to the respective lengths of each of these contrasting organs (e.g. long nectary spurs can only be accessed by insects with a long proboscis—Whittall and Hodges 2007). *Utricularia* spp. show great variation in the size and, in particular, the length of their nectary spurs (Taylor 1989). Both species investigated here, as well as *U. reniformis*, possess large and relatively long spurs which can be accessed easily by long-tongued bumblebees, as has been demonstrated for this last taxon (Clivati et al. 2014). Peltate glandular trichomes are present in the nectary spurs of *U. cornigera* and *U. nelumbifolia* and, in *U. nelumbifolia*, are arranged in two tracts coinciding and seemingly closely associated with the main vascular bundles supplying the apical part of the nectary spur. This suggests that sugars are translocated in the phloem directly to the point of nectar secretion (Nepi 2007). Furthermore, observations reported here indicate that the cuticle overlying the heads of these trichomes becomes distended in response to the subcuticular accumulation of nectar, as what occurs in the nectaries of other unrelated taxa (Nepi 2007 and references therein). The trichomes lining the spur of *U. cornigera* and *U. nelumbifolia* (comprising a basal cell, a central cell whose walls stain selectively with Sudan stains and may thus act as a hydrophobic barrier and a multicellular head) are typical of the genus and occur in all three subgenera (*Polypompholyx*, *Bivalvaria* and *Utricularia* sensu Müller and Borsch 2005), as well as various sections, such as *Pleiochasia* (*U. dichotoma*, *U. paulinae*, *U. dunlopii—*Płachno et al. 2015), *Oligocista* (*Utricularia arcuata—*Farooq 1963; *U. reticulata*, *Utricularia scandens*—Farooq and Siddiqui 1966), *Utricularia* (*Utricularia gibba*, *Utricularia inflata*—Farooq and Siddiqui 1966; *U. inflexa*—Farooq 1963), *Vesiculina* (*Utricularia purpurea*—Farooq and Siddiqui 1966) and *Iperua* (*U. reniformis*—Clivati et al. 2014; *U. cornigera* and *U. nelumbifolia*—the subjects of the present paper). Thus, it would appear that nectar-secreting trichomes in the genus *Utricularia* are very conservative in evolutionary terms.

Unfortunately, information concerning the micro-morphological features of *Utricularia* spurs is scarce. According to Clivati et al. (2014), epidermal papillae occur in the nectary spur of *U. reniformis*, and recently, we recorded for the nectary spur of *U. dunlopii*, unicellular papillae containing starch-laden plastids. The cuticle of these cells contained numerous micro-channels (Płachno et al. 2015). Small, unicellular papillae were also found in the nectary spurs of *U. dichotoma*, *U. paulinae* and *U. uniflora* (Płachno et al. 2015). The presence of conical papillae lining the nectary spur of this genus, each having a cuticle that is striate and containing numerous micro-channels, indicates that these papillae may participate in nectar reabsorption, as has been proposed for certain orchids (Stpiczyńska 2003; Bell et al. 2009).

### Ecological considerations

*U. nelumbifolia* and *U. cornigera* have zygomorphic and ‘gullet-shaped’ flowers that closely resemble those of *U. reniformis* both morphologically and also in terms of micro-morphology. According to Faegri and van der Pijl (1971), the reproductive structures of ‘gullet-shaped’ flowers are located dorsally, and thus, pollen is deposited on the back of the pollinator (upper side of the head). This pattern seems to be typical of the members of Lamiales. These also have strongly zygomorphic flowers, whose lower lip presents a landing area for hovering pollinators. The pollinators of *U. reniformis* are large bees (Clivati et al. 2014) of the genera *Bombus* and *Xylocopa*, and these are able to prise open the flower, exposing the stamens and stigma that occur beneath the upper lip of the corolla. Smaller insects are not sufficiently strong to do this and cannot enter the flower. Even if they could, they would be too small to reach the reproductive organs. *U. cornigera* and *U. nelumbifolia* are sympatric, and since their flowers so closely resemble those of *U. reniformis*, all three species probably share the same pollinators.

*U. nelumbifolia* occurs inside the urns of species of the bromeliad genus *Vriesea* Lindl. that grows on the vertical cliffs of inselbergs—a very specific habitat that can be colonized by few plant species. As a result, characters that enable potential pollinators to locate and identify the flowers of these species projecting from dense populations of bromeliads on vertical slopes are important. Not only does *U. cornigera* grow in the urns of *Vriesea*, but also from the leaf rosettes of *Eryngium* L. (Apiaceae—Studnička 2011). This combination of large, colourful, obvious and seemingly fragrant (though odourless to humans), nectariferous flowers, whose bilabiate corolla can probably only be prised open by large bees, coupled with the fact that these plants are associated with a very specialized ecological niche (as an aquatic epiphyte on the vertical cliffs of inselbergs) may be key to the ecological and evolutionary success of these species.

It is worth to mention that *Utricularia* species from sect. *Iperua* and sect. *Orchidioides* are orchid-like bladderworts. There are many similar characters in habitat (lithophyte and epiphytic species), morphology and anatomy (e.g. occurrence of water storage organs) and the seed structure; thus, this is an example of parallel evolution (e.g. Taylor 1989; Juniper et al. 1989; Adlassnig et al. 2005). Orchid seedlings are dependent on fungi, whereas *Utricularia* seedlings are dependent on caught prey.

### Conclusions

Epidermal structures borne on the palates of the two species investigated here have the potential to provide a range of stimuli. These may be visual, tactile and probably also olfactory and may have both the capacity to attract and regulate the behaviour of potential insect pollinators. The data now shows that these plates are unguentarii as far as current technology allows. Final proof can only come with advances in our ability to detect very small amounts of volatiles excitatory for non-humans. Both species possessed nectar-secreting trichomes similar to those found in other species of *Utricularia*, and there was evidence that the cuticle of the multicellular head of the trichome became distended, as what occurs in many species of nectariferous orchid, as nectar accumulates beneath its surface. Thus, in evolutionary terms, the micro-morphology of the nectary spur of *Utricularia* is rather conservative, and on the basis of gross floral morphology and ecological studies, it is proposed that flowers of *U. cornigera* and *U. nelumbifolia* are bee-pollinated.
